# The genomic characteristics of RET fusion positive tumors in Chinese non-small cell lung cancer (NSCLC) patients

**DOI:** 10.1007/s00432-022-03959-6

**Published:** 2022-02-26

**Authors:** Guowu Wu, Longhua Guo, Yinfang Gu, Tanxiao Huang, Ming Liu, Xiaofang Zou, Bo Yang, Ping Huang, Chunling Wen, Lilan Yi, Wenting Liao, Dongdong Zhao, Junlin Zhu, Xiaoni Zhang, Yuanyuan Liu, Yan Yin, Shifu Chen

**Affiliations:** 1 Department of Medical Oncology, Cancer Center, Meizhou People’s Hospital (Huangtang Hospital), 63 Huangtang Road, Meizhou, China; 2 HaploX Biotechnology Co., Ltd., Shenzhen, China

**Keywords:** RET, Non-small cell lung cancer, Next generation sequencing

## Abstract

**Background:**

Approximately 1–2% of non-small cell lung cancer (NSCLC) patients harbor RET (rearranged during transfection) fusions. The oncogenic RET fusions could lead to constitutive kinase activation and oncogenesis.

**Methods:**

1746 Chinese NSCLC patients were analyzed in this study. Tumor tissues were collected, and were formalin fixed, paraffin-embedded (FFPE) and archived. Peripheral blood (PB) samples were also collected from each patient as control. In addition, we selected 17 of them for cfDNA NGS testing and 14 tumor samples for immunohistochemistry testing using PD-L1 rabbit monoclonal antibody, clones 28-8 (Abcam, Cambridge, UK).

**Results:**

Of the 1746 NSCLC cases, RET rearrangements were identified in 25 cases (1.43%) with locally advanced or metastatic NSCLC, of which 20 (80%) were female. We found that 14 out of 25 patients had an KIF5B-RET fusion, with KIF5B exon15-RET exon12, KIF5B exon23-RET exon12, and KIF5B exon24-RET exon11 detected in 14, 3, and 1 patients, respectively. We also identified one novel RET fusion partner PLCE1 and 4 intergenic-breakpoint fusions.

**Conclusion:**

In this study, using the hybrid capture based next generation sequencing (NGS) techniques, we revealed the genomic profiling for the patients with RET fusion-positive NSCLC. To the best of our knowledge, this is the first study that exhibited the detailed breakpoints of Chinese NSCLC patients with RET rearrangement, and we found a novel new partner PLCE1. The results provided genomic information for patients with RET fusion which is significant for personalized clinical management in the era of precision medicine.

## Background

It was not until 2012 that the RET fusions were identified as oncogenic drivers in NSCLC (Ju et al. 2012a). Approximately 1–2% of NSCLC patients harbor RET fusions (Cancer Genome Atlas Research Network 2014), and they tend to be young, non-smokers, and adenocarcinomas (Wang et al. 2012). The RET proto-oncogene encodes a transmembrane receptor tyrosine kinase belonging to members of the glial cell line-derived neurotrophic factor (GDNF) family. The RET signaling is modulated by ligands and could activate multiple downstream pathways, such as RAS/MAPK/ERK, PI3K/AKT and JAK/STAT, which are essential for cellular differentiation and proliferation. However, the oncogenic RET fusions could lead to constitutive kinase activation and oncogenesis (Worby et al. 1996; Qian et al. 2014; Trupp et al. 1999). RET fusions are caused by chromosomal rearrangement, which fuses the 3′ coding regions for the RET kinase domain on chromosome 10 with a 5′ upstream partner gene containing coiled-coil or LISI homology domains (Kohno et al. 2012; Lipson et al. 2012; Ju et al. 2012b). The most common partner genes are KIF5B, CCDC6 and NCOA4, which originate from intrachromosomal rearrangements, but there are also some relatively infrequent interchromosomal partner genes, such as TRIM33, TRIM24, MYO5C, EPHA5, CLIP1, and so on (Chao et al. 2012; Ferrara et al. 2018).

The advent of precision medicine has revolutionized the therapeutic landscape of NSCLC, and targeted therapies have been investigated in many clinical studies against patients with RET fusion-positive NSCLC, which includes multi-kinase inhibitors (MKIs) and selective RET inhibitors (Choudhury and Drilon 2020). MKIs target not only RET, but also other kinases, such as VEGFR2, KIT, BRAF, etc. This probably leads to decreased effectiveness against RET and off-target side effects. Several MKIs that were approved by FDA for cancer therapy on other purposes (such as sunitinib, sorafenib, vandetanib, cabozantinib, regorafenib, lenvatinib, and alectinib) all showed modest clinical activity and had side effects for patients with RET fusion-positive NSCLC (Drilon et al. 2016, 2018, 2019; Gautschi et al. 2017; Yoh et al. 2017; Ribeiro et al. 2020). Besides, MKI resistance is almost inevitable. The common resistant mechanisms are secondary MET gene mutation and downstream signaling pathway activation (Nakaoku et al. 2018; Nelson-Taylor et al. 2017). These limitations in MKIs prompt the development of selective RET inhibitors. On May 8, 2020, the FDA granted accelerating approval to selpercatinib for adult patients with metastatic RET fusion-positive NSCLC. This was the first targeted therapy approved for RET fusion-positive NSCLC (Markham 2020). The other selective RET inhibitor, pralsetinib, was also granted as Breakthrough Therapy designation by the FDA for advanced NSCLC with RET fusions after progression on platinum chemotherapy. Both of them showed higher response rates and tolerability, but drug resistance were also inevitable for them (Velcheti et al. 2017; Subbiah et al. 2018).

The development of hybrid capture-based NGS techniques brings great convenience in revealing the genomic profiling for cancer patients. It’s becoming more and more available in clinical cancer treatment. It not only could be used to identify the RET fusion events, but also allows us to investigate the co-occurring genomic alterations in the same assay, which may be related to prognosis or therapeutic response. In this study, we sequenced 1746 NSCLC patients by hybrid capture-based NGS techniques, and eventually identified 25 RET fusion-positive cases.

## Methods

### Patients and samples

In this study, 1746 Chinese NSCLC patients were analyzed. Tumor tissues and peripheral blood (PBL) samples were collected for each patient. Tumor tissues were formalin fixed, paraffin-embedded (FFPE), while PB samples were tested as control. In addition, we selected 17 of them for cfDNA NGS testing and 14 tumor samples for immunohistochemistry testing using PD-L1 rabbit monoclonal antibody, clones 28-8 (Abcam, Cambridge, UK).

### FFPE DNA extraction

DNA samples from tumor tissues were extracted using QIAamp DNA FFPE tissue kit (Qiagen) after paraffin-embedded. DNA samples from PBL were extracted using the RelaxGene Blood DNA system (Tiangen Biotech Co., Ltd., Beijing, China). Quantification of all the DNA samples were conducted by both the Qubit 2.0 fluorometer and the Qubit dsDNA HS Assay kit (Thermo Fisher Scientific, Inc., Waltham, MA, USA).

### Plasma isolation and cfDNA extraction

Blood samples from patients were collected in tubes containing EDTA and centrifuged at 1600*g* for 10 min at 4 °C within 2 h of collection. The peripheral blood lymphocyte (PBL) debris was stored at − 20 °C until further use. The supernatants were further centrifuged at 10,000*g* for 10 min at 4 °C, and plasma was harvested and stored at − 80 °C until further use. DNA from PBLs was extracted using RelaxGene Blood DNA System (TianGen Biotech Co., Ltd., Beijing, China), and cell free DNA (cfDNA) was extracted from at least 2 mL plasma using QIAamp Circulating Nucleic Acid kit (QIAGEN) following the manufacturers’ instructions, respectively. Extracted DNA was then quantified by Qubit 2.0 (Thermo Fisher Scientific, Inc., Waltham, MA, USA), according to manufacturer's instructions.

### FFPE and genomic DNA library construction and sequencing

100 ng of FFPE DNA and genomic DNA from PBLs for each patient was sheared by the dsDNA Fragmentase (New England BioLabs, Inc., Ipswich, MA, USA). And then, size of 150–250 bp were selected using Ampure XP beads (Beckman Coulter, Inc., Brea, CA, USA). Library construction was conducted using the KAPA Library Preparation kit (Kapa Biosystems, Inc., Wilmington, MA, USA). And then, the concentration assessment of the library was performed using the Qubit dsDNA HS Assay kit, while the fragment length was acquired on a 4200 Bioanalyzer (Agilent Technologies, Inc., Santa Clara, CA, USA). Targeted capture was performed using a set of customized biotinylated DNA probes (HapOncoCDx panel) which contained 464 cancer-related genes encompassing 1.31 Mb (Roche NimbleGen). The hybridization of the amplified sample libraries and the SeqCap EZ Library was used according to the manufacturer’s protocol for 16–20 h at 47 °C. After hybrid selection, the captured DNA fragments were amplified with 12–14 cycles of PCR using 1 × KAPA HiFi Hot Start Ready Mix and Post-LM-PCR Oligos in two separate 50 μL reactions. The reactions were then pooled and purified by Agencourt AMPure XP beads. DNA sequencing was then performed using Illumina Novaseq 6000 system with an average depth at 2000X.

### CfDNA library construction and sequencing

Library construction was conducted using cfDNA with the KAPA Library Preparation kit (Kapa Biosystems, Inc., Wilmington, MA, USA). Agencourt AMPure XP beads (Beckman Coulter, Inc., Brea, CA, USA) were applied for cleanup steps. DNA fragments were purified using the Qubit dsDNA HS Assay kit and the concentration was evaluated by the Qubit 2.0 fluorometer. Then end repair and 3′-end A-tailing were conducted. Ligation was performed at 20 °C for 15 min. Single-step size selection was achieved by 50 μL (1 ×) of PEG/NaCl SPRI Solution buffer. Then the ligated fragments were amplified in 1 × KAPA HiFi Hot Start Ready Mix with Pre-LM-PCR Oligos in 50 μL reactions, thereafter PCR were performed with 7–12 cycles depending on the quantity of input DNA. Qubit 2.0 Fluorometer and Qubit dsDNA HS Assay kit were applied again to evaluate the library’s purity and concentration. Fragment length was detected on a 4200 Bioanalyzer using DNA 1000 Kit (Agilent).

Targeted capture was conducted using customized HapOncoCDx panel. The hybridization of the amplified sample libraries and the SeqCap EZ Library was used according to the manufacturer’s protocol for 16–20 h at 47 °C. After hybrid selection, the captured DNA fragments were amplified in PCR reaction using 1 × KAPA HiFi Hot Start Ready Mix and Post-LM-PCR Oligos with 12–14 cycles. The reactions were then purified by Agencourt AMPure XP beads. Multiplexed libraries were denatured by Tris–HCl and diluted by 0.2 N NaOH according to the manufacturer’s protocol (Illumina). Then the libraries were sequenced using 150-bp paired-end runs on an Illumina NovaSeq 6000 system (Illumina).

### Data analysis and variant calling

Raw data were pre-processed by fastp with version 0.18.0 (https://github.com/OpenGene/fastp) (Chen et al. 2018a). Then clean reads were aligned to hg19 genome (GRch37) using maximal exact matches algorithm of Burrows–Wheeler Aligner (Li and Durbin 2010). Duplicate reads were removed by Gencore version 0.12.0 (https://github.com/OpenGene/gencore) (Chen et al. 2021). After applying Samtools version 0.1.19 (http://www.htslib.org/) (Li et al. 2009), pileup files were generated with mapping quality ≥ 60. VarScan2 with version 2.3.8 (http://varscan.sourceforge.net/) (Koboldt et al. 2012) were applied to call somatic variants [minimum read depth = 20; variant allele frequency (VAF) threshold ≥ 0.01; somatic-*P* value ≤ 0.01; strand-filter = 1; other parameters, default]. Copy number variation were detected using CNV kit with version 0.9.3 (https://github.com/etal/cnvkit) (Talevich et al. 2014), while structural variation were calculated by GeneFuse with version v0.6.1 (https://github.com/OpenGene/GeneFuse) (Chen et al. 2018b). Microsatellite instability (MSI) status were determined by VisualMSI (Chen et al. 2019). Maftools were used for visualizing somatic variant analysis (Mayakonda et al. 2018). Data which met the following criteria were chosen for subsequent analysis: the ratio of remaining data filtered by fastq in raw data is ≥ 85%; the proportion of Q30 bases is ≥ 85%; the ratio of reads on the reference genome is ≥ 85%; target region coverage ≥ 98%; average sequencing depth in tissues is ≥ 500 ×; average sequencing depth in blood cfDNA is ≥ 1000 ×. The called somatic variants need to meet the following criteria: the read depth at a position is ≥ 100 ×; the variant allele frequency (VAF) is ≥ 2% for tissue DNA and ≥ 0. 2% for cfDNA from blood; somatic-*P* ≤ 0.01; strand filter = 1. Allele frequencies were calculated for Q30 bases. For cfDNA, somatic variant calls (SNV or indel) present at least on 5 unique reads, at least 1 on each strand, and less than 0.5% mutant allelic frequency in the paired normal sample (PBLs) were retained. A manual visual inspection step was used to further remove artificial changes by GenomeBrowse (GenomeBrowse 2021).

## Results

### Sample collection and patient characteristics

The clinical information of all the 1746 patients are summarized in Table 1. Of the 1746 NSCLC cases, RET rearrangements were identified in 25 cases (1.43%) with locally advanced or metastatic NSCLC, of which 20 (80%) were female. The 25 patients have mean age of 53.5 ranging from 27 to 78. All of the 25 patients had lung adenocarcinoma. NGS testing was performed on their 25 pairs of FFPE tumor tissue and PBL samples. In addition, we performed cfDNA NGS testing on 17 of them to check their RET gene status in cfDNA sample. All the samples passed the histology quality control (HQC) and yielded sufficient DNA for NGS.

**Table 1:** Summary of patients

	Number
*N*	1746
Age (median [P_25_, P_75_])	59.50 [51.00, 67.00]
Sex (%)	
Female	811(46.5)
Male	935(53.6)
Classification (%)	
Adenocarcinoma	1503 (86.1)
Squamous	216 (12.4)
Unknown	27 (1.5)
Stage at diagnosis (%)	
I, II	639(36.6)
III, IV	1042(59.7)
Unknown	65(3.7)

### Identification of RET rearrangements using targeted sequencing

**Fig. 1:**
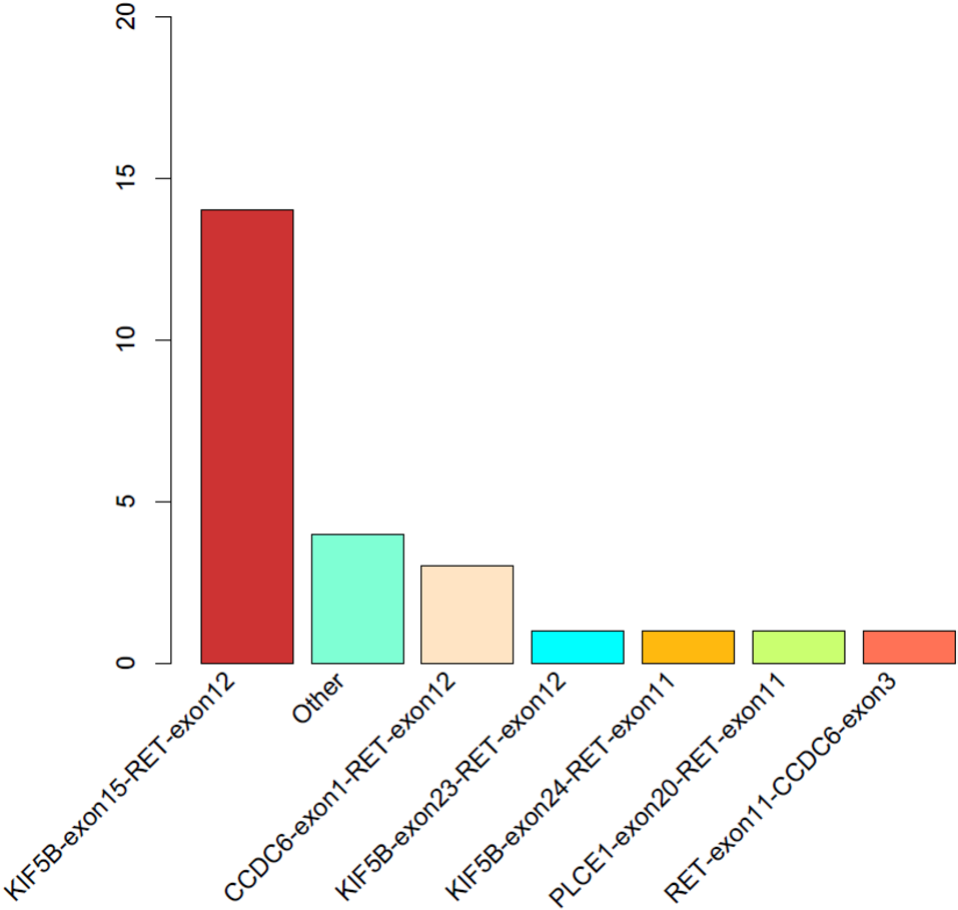
Statistics of different RET rearrangement forms. The distribution of each RET fusion pattern identified in 25 NSCLC patients are shown in the barchart

**Fig. 2:**
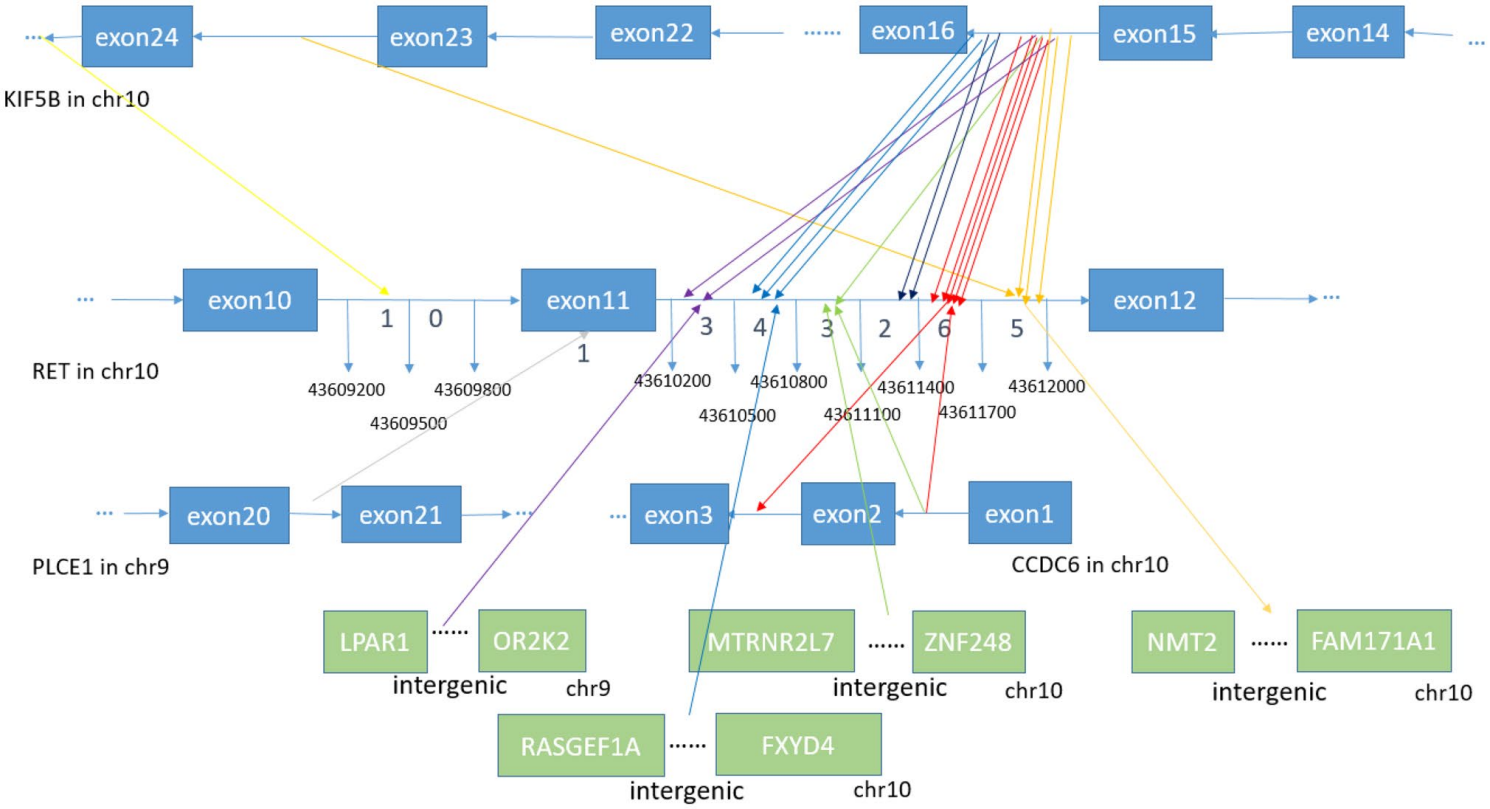
Breakpoint distribution in RET and the corresponding fusion partners. Each fusion event was represented with an arrowed line. The breakpoints of RET in GRch37 was shown in the middle panel with the fused exons of RET fusion partners on the top or bottom panels. The sequences of KIF5B and CCDC6 were exhibited reversely (from right to left), while the sequences of RET and PLCE1 were represented in the forward direction. The genomic region of RET between 43,609,200 and 43,609,800 in intron 10 as well as region between 43,610,200 and 43,612,000 in intron 11 was divided into regions every 300 bp. Breakpoint positions in RET located between 43,609,200 and 43,609,500 with an yellow arrow, between 43,610,200 and 43,610,500 with a purple arrow, between 43,610,500 and 43,610,800 with a blue arrow, between 43,610,800 and 43,611,100 with an green arrow, between 4,361,100 and 43,611,400 with a black arrow, between 4,361,400 and 43,611,700 with a red arrow, between 4,361,700 and 43,612,000 with an orange arrow

In this study, we designed probes to cover the intron 6, 7, 8, 9, 10, 11 of RET as well as introns of some well-known RET fusion partners to identify RET rearrangement of the DNA from patients’ FFPE samples. We identified RET rearrangements and analyzed the corresponding breakpoints for these patients. The statistical summary of the rearrangement events is presented in Table 2 and Fig. 1. The breakpoint distribution in RET is shown in Fig. 2. The results showed that 14 out of 25 patients had an KIF5B-RET fusion, with KIF5B exon15-RET exon12, KIF5B exon23-RET exon12, and KIF5B exon24-RET exon11 detected in 14, 3, and 1 patient, respectively. It also detected one novel RET fusion partner PLCE1 and 4 intergenic-breakpoint fusions.

**Table 2:** Fusion patterns of RET

Fusion type	Counts	Percent (%)
KIF5B-exon15-RET-exon12	14	56
CCDC6-exon1-RET-exon12	3	12
KIF5B-exon23-RET-exon12	1	4
KIF5B-exon24-RET-exon11	1	4
PLCE1-exon20-RET-exon11	1	4
RET-exon11-CCDC6-exon3	1	4
Other	4	16

### Mutational profiles of RET fusion-positive NSCLC patients

**Fig. 3:**
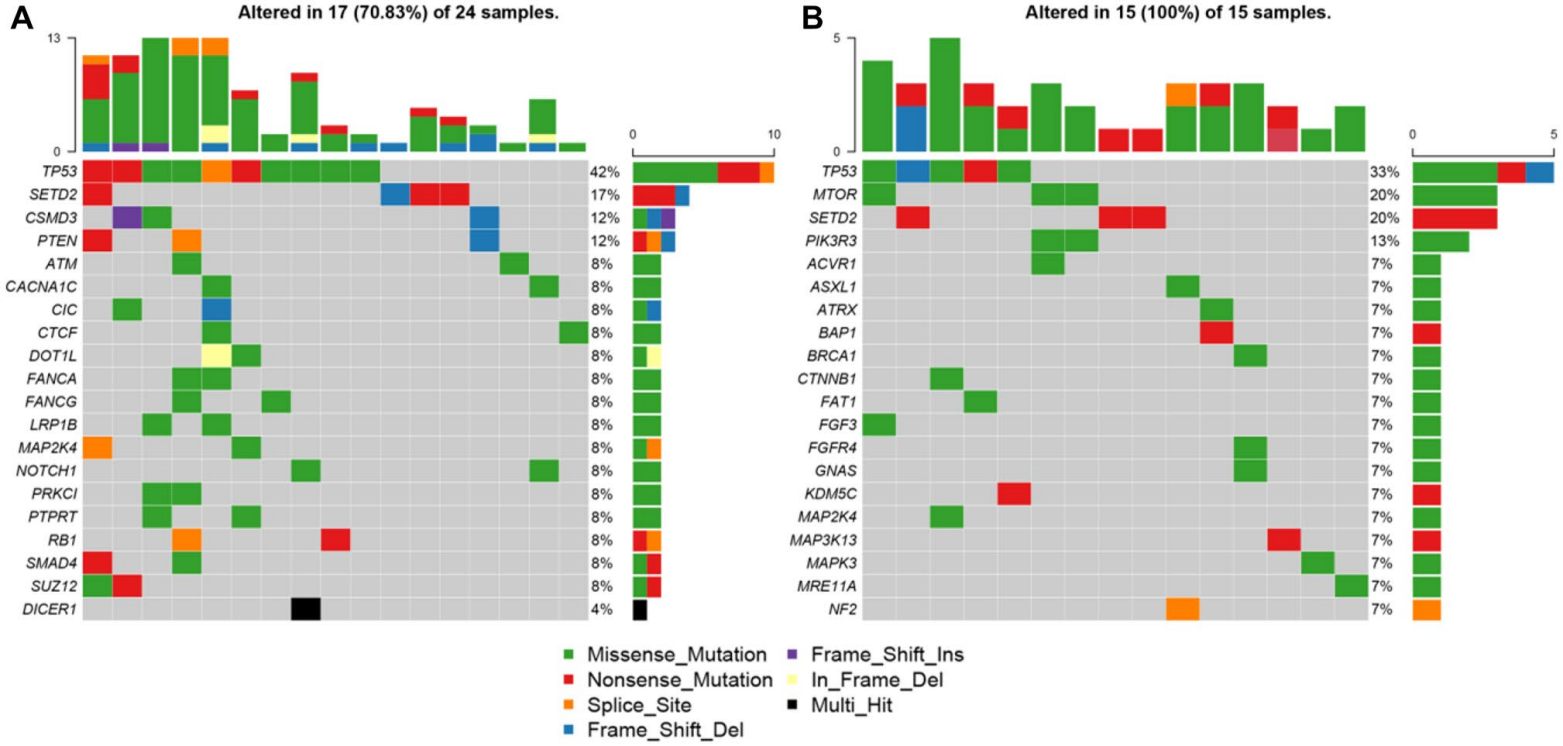
Mutational profiles of RET fusion-positive NSCLC patients. **A** The oncoprint for the top 20 genes of the somatic SNVs and Indels of the 25 patients in our study. Somatic alterations included missense, nonsense, frameshift indel, in-frame indel, splice site, translation start site, and multi_Hit mutations. The genes were ranked by the frequency of mutations across all samples. **B** The oncoprint for the top 20 genes of the somatic SNVs and Indels of the 15 patients from the MSK-IMPACT study (Zehir and Benayed 2017). Somatic alterations included missense, nonsense, frameshift indel, in-frame indel, and splice-site mutations. The genes were ranked by the frequency of mutations across all samples

Genomic alterations were detected in 24 (*n* = 24/25, 96%) samples with a total of 113 alterations including nonsynonymous mutations and splicing mutations. The top 20 alterations are listed in Fig. 3A. The mutation landscapes of RET fusion-positive NSCLC patients were highly heterogeneous. The median TMB was 2.4 mut/Mb with a range between 0 to 8.4 mut/Mb, which is similar to the TMB value of TCGA NSCLC cohort (Chalmers et al. 2017).

Besides, a heatmap was created to illustrate the somatic mutations detected in the tumor tissues of the patients (Fig. 3A). TP53 was the top altered (*n* = 10, 42%), followed by SETD2 (*n* = 4, 17%), CSMD3 (*n* = 3, 12%), and PTEN (*n* = 3, 12%). Other genomic alterations with low frequencies were ATM (*n* = 2, 6%), CACNA1C (*n* = 2, 8%), CIC (*n* = 2, 8%), CTCF (*n* = 2, 8%), DOT1L (*n* = 2, 8%), FANCA (*n* = 2, 8%), FANCG (*n* = 2, 8%),LRP1B (*n* = 2, 8%), MAP2K4 (*n* = 2, 8%), NOTCH1(*n* = 2, 8%), PRKCI (*n* = 2, 8%), PTPRT (*n* = 2, 8%), RB1 (*n* = 2, 8%), SMAD4 (*n* = 2, 8%), and SUZ12 (*n* = 2, 8%). Alterations in DICER1 were identified in one sample (*n* = 1, 4%). Moreover, the results were compared with the MSK-IMPACT study (Mayakonda et al. 2018), from which we extracted 30 RET fusion positive cases that yielded 81 mutations. Overall, the results of our study and MSK-IMPACT research were highly consistent, both of which showed that TP53 and SETD2 were the most frequently altered genes (Fig. 3B).

**Fig. 4:**
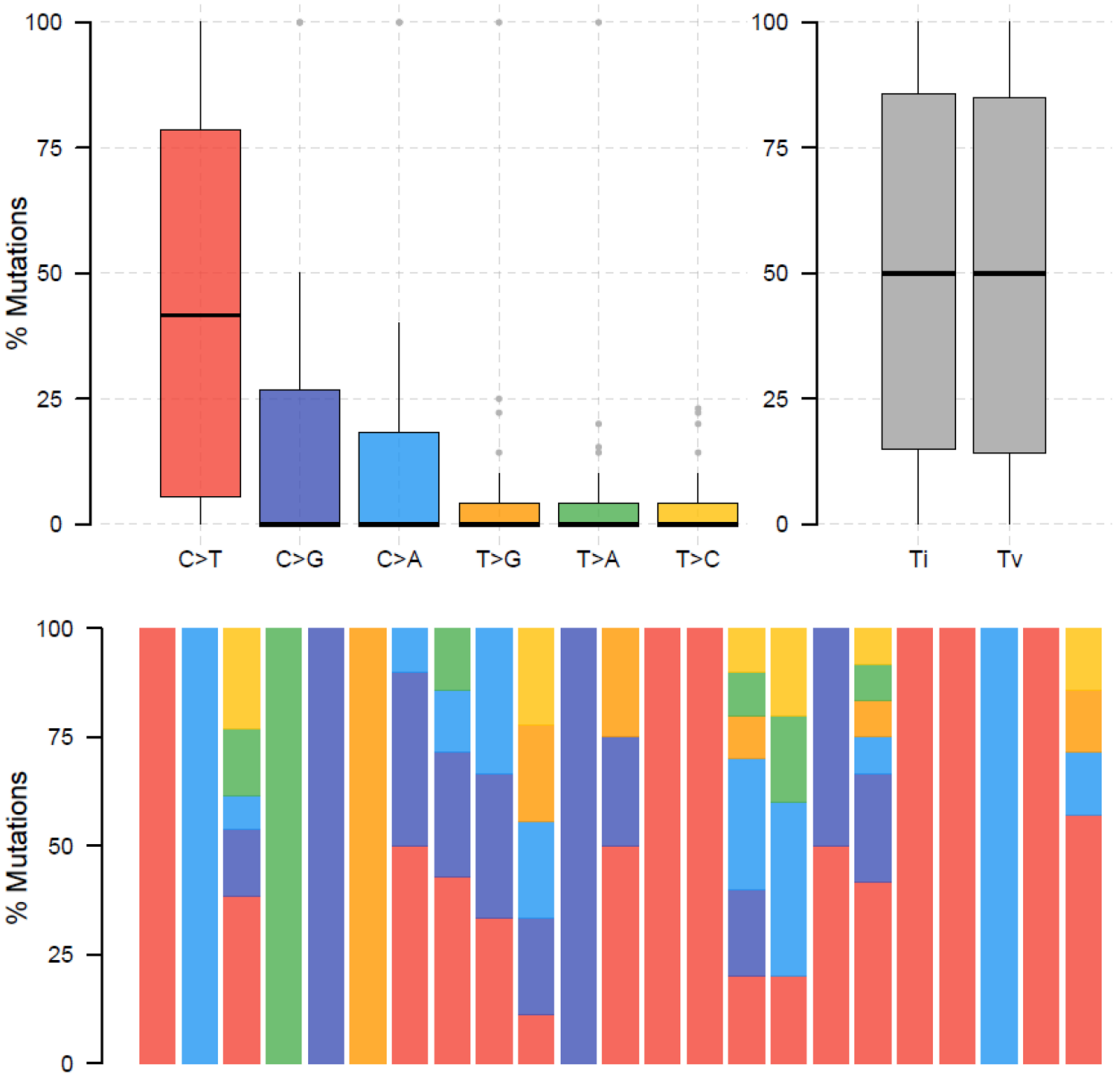
Mutational signatures of RET fusion positive NSCLC patients. SNPs are summarized into transitions and transversions. Statistical data were visualized as a boxplot presenting overall distribution of six kinds of different conversions (top) as well as a stacked barplot presenting the fraction of different kinds of conversions in each sample (bottom)

Then the mutational signatures were further studied. It was observed that C>T transition happened most frequently, followed by C>G transversions (Fig. 4). This pattern is consistent with COSMIC signature 84 according to website (https://cancer.sanger.ac.uk/signatures/sbs/sbs84/) that had been found in some cancer samples.

**Fig. 5:**
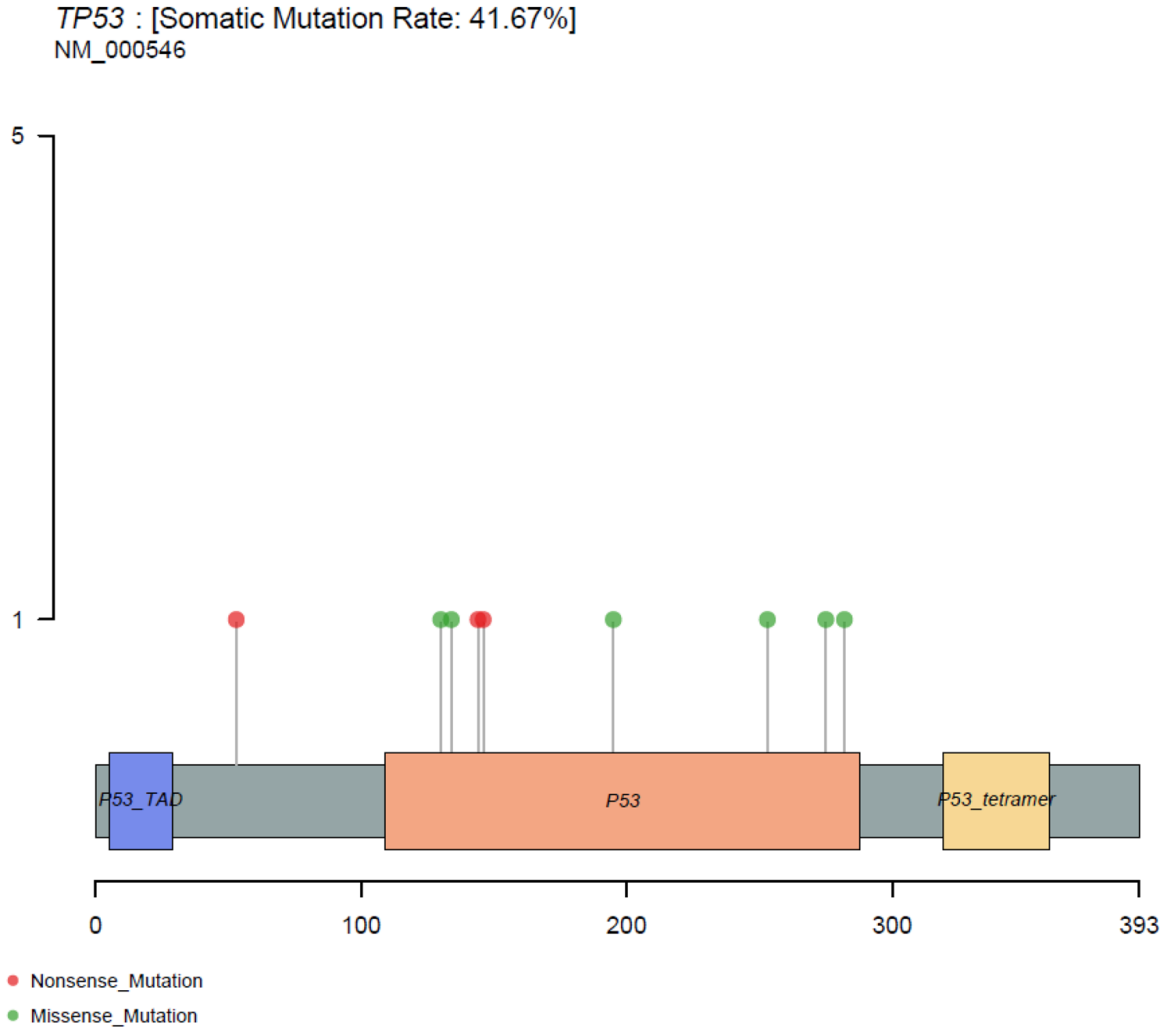
Protein variants resulted from TP53 mutations. The lollipop plot showed the protein variants caused by TP53 mutations, which were considered to be mutational hot-spots

Different driver gene mutations demonstrated inter-tumor heterogeneity. TP53 mutations in exon 4–8 were observed, and the TP53 mutation sites on the peptide sequence were elaborately portrayed in a lollipop plot (Fig. 5).

### Copy number aberrations of RET fusion-positive NSCLC patients

**Fig. 6:**
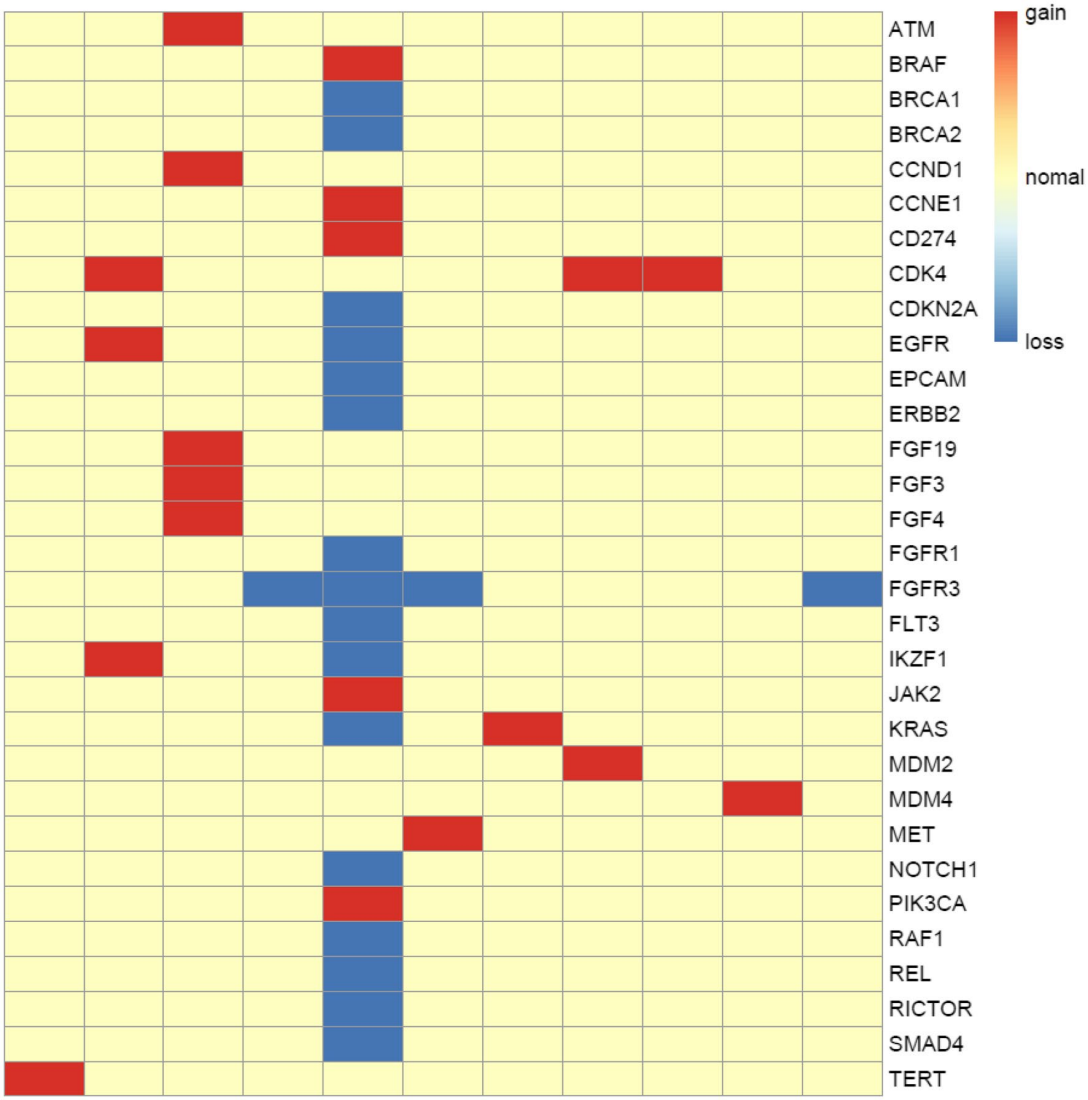
Copy number aberrations in 11 RET fusion-positive NSCLC patients. The *y*-axis represented the names of the aberrant genes, while *x*-axis represents individual patient. The types of copy number aberrations, including gain, normal and loss were indicated by red, yellow, and blue, respectively

Somatic copy number alterations were detected in 11 (*n* = 11/25, 44%) samples. A total of 22 alterations were discovered, including gain and loss (Fig. 6). CDK4 were the most commonly amplified gene (*n* = 3/11, 27%). Loss of copy number was observed in FGFR3 with highest frequency (*n* = 4/11, 36%).

### PD-L1 expression and microsatellite Instability (MSI) status of RET fusion-positive NSCLC patients

High (≥ 50%), intermediate (1–49%), and negative (< 1%) PD-L1 expression was observed in 0/14 (0%), 8/14 (57%), and 6/14(43%) cases, respectively.

MSI status were evaluated in 20 cases. They were all microsatellite stable (MSS).

### Concordance in tumor DNA (tDNA) and plasma DNA (ctDNA) sample pairs of RET fusion-positive NSCLC patients

A total of 17 tDNA and ctDNA sample pairs were analyzed. 9 patients are found to have the same breakpoint of RET fusions in both tDNA and ctDNA samples, indicating 52.9% RET fusion could be detected in ctDNA. A total of 111 mutations (snv and indel) were identified, including 90 in tDNA and 64 in plasma ctDNA, and 43 concordant mutations in both tDNA and plasma ctDNA. Seven sample pairs (7/17, 41.2%) had concordant mutations in both tDNA and plasma ctDNA, and the average variant frequency in these plasma ctDNA samples was 8.17%.

## Discussion

In this study, we identified RET rearrangement events in 25 Chinese NSCLC patients by hybrid capture based NGS. Consistent with other reports, the most common RET fusion partner was KIF5B and CCDC6, and the breakpoints in the genome mainly located in the intron 11 of RET, intron 15 of KIF5B, and intron 1 of CCDC6. Amazingly, we discovered a new RET fusion partner PLCE1. Besides, we identified 4 intergenic-breakpoint fusions in 4 cases. A study by Weihua Li reported that intergenic-breakpoint fusions might also generate functional fusion transcripts (Li et al. 2020), so additional validation testing such as RNA-seq or IHC was required for these patients to guide treatment. At the same time, we checked the concordance of RET fusions between tDNA and cfDNA for the same patient. In most cases, they harbored the same breakpoint, this clearly proved that the fragments of DNA harboring RET fusions were derived from tDNA. Therefore, cfDNA is an excellent alternative material for patients who have difficulty in obtaining tumor tissues. These results also implied that NGS-based assessment for RET fusions had the advantages of detecting unknown RET fusion partners and identifying the same breakpoints as the traditional diagnostic testing, such as FISH and IHC.

At the same time, we characterized the co-occurring genomic alterations of these RET fusion-positive patients. The results were consistent with the TCGA cohort, in terms of the relatively higher frequency of TP53 mutations, fewer co-mutations, and lower TMB compared to RET fusion-negative NSCLC patients. Moreover, we analyzed the copy number alterations in the genome of the patients. Besides the genes with frequent copy number amplification, such as CDK4, we also discovered some genes with frequent copy number loss, such as FGFR3. This information was important for guiding optimal clinical treatment.

## Conclusion

In conclusion, we successfully detected the RET fusion events in 25 Chinese NSCLC patients using our customized HapOncoCDx panel. In addition, we also explored the genomic mutational landscapes of the patients. This is the first study that explored the details of breakpoints for Chinese NSCLC patients with RET rearrangement, and we discovered a novel new partner PLCE1. The results provided genomic information for patients with RET fusion which is significant for personalized clinical management in the era of precision medicine.

## Data Availability

The data sets for this manuscript are not publicly available due to data privacy. Requests to access the data sets should be directed to the corresponding authors Guowu Wu (guowuwugd@163.com), Longhua Guo (1851258807@qq.com), and Shifu Chen (chen@haplox.com).

## Abbreviations

NSCLC: Non-small cell lung cancer
MKIs: Multi-kinase inhibitors
FFPE: Formalin-fixed paraffin-embedded
PBL: Peripheral blood lymphocyte
cfDNA: Cell free DNA
MSI: Microsatellite Instability
